# P.F508del editing in cells from cystic fibrosis patients

**DOI:** 10.1371/journal.pone.0242094

**Published:** 2020-11-11

**Authors:** Svetlana A. Smirnikhina, Ekaterina V. Kondrateva, Elmira P. Adilgereeva, Arina A. Anuchina, Milyausha I. Zaynitdinova, Yana S. Slesarenko, Angelina S. Ershova, Kirill D. Ustinov, Matvei I. Yasinovsky, Elena L. Amelina, Ekaterina S. Voronina, Valentina D. Yakushina, Vyacheslav Yu. Tabakov, Alexander V. Lavrov

**Affiliations:** 1 Laboratory of Genome Editing, Research Centre for Medical Genetics, Moscow, Russian Federation; 2 Nazarbayev University, School of Science and Technology, Nur-Sultan, Republic of Kazakhstan; 3 Laboratory of Cystic Fibrosis, Research Institute of Pulmonology, Moscow, Russian Federation; Lewis Katz School of Medicine at Temple University, UNITED STATES

## Abstract

Development of genome editing methods created new opportunities for the development of etiology-based therapies of hereditary diseases. Here, we demonstrate that CRISPR/Cas9 can correct p.F508del mutation in the *CFTR* gene in the CFTE29o- cells and induced pluripotent stem cells (iPSCs) derived from patients with cystic fibrosis (CF). We used several combinations of Cas9, sgRNA and ssODN and measured editing efficiency in the endogenous *CFTR* gene and in the co-transfected plasmid containing the *CFTR* locus with the p.F508del mutation. The non-homologous end joining (NHEJ) frequency in the *CFTR* gene in the CFTE29o- cells varied from 1.25% to 2.54% of alleles. The best homology-directed repair (HDR) frequency in the endogenous *CFTR* locus was 1.42% of alleles. In iPSCs, the NHEJ frequency in the *CFTR* gene varied from 5.5% to 12.13% of alleles. The best HDR efficacy was 2.38% of alleles. Our results show that p.F508del mutation editing using CRISPR/Cas9 in CF patient-derived iPSCs is a relatively rare event and subsequent cell selection and cultivation should be carried out.

## Introduction

Cystic fibrosis (CF) is a severe systemic monogenic disease resulting from an imbalance of chloride ions. The disease is caused by mutations in the cystic fibrosis transmembrane conductance regulator (*CFTR*) gene which encodes the CFTR chloride ion channel [1]. The ion channel expressed at the apical membrane of epithelial cells is involved in the transport of chloride ions as well as bicarbonates [2]. The p.F508del mutation (phenylalanine deletion at position 508) is most common in CF patients [3].

For many years, symptomatic therapy has been the only available treatment for CF. The main objectives of such therapy are the treatment of pulmonary infections (antibiotics and anti-inflammatory drugs, bronchodilators, mucolytics, and expectorant drugs) and the malabsorption syndrome (enzyme replacement therapy to normalize the function of the gastrointestinal tract) [4]. However, despite the obvious successes of symptomatic therapy, it does not completely stop the progression of the disease.

Owing to the development of CFTR modulators in 2009, cystic fibrosis turned from a disease with a high infant mortality rate into a disease with a predicted median survival of 43.6 years [3]. Pathogenesis-based therapy includes correctors of protein folding and processing (lumacaftor [5], tezacaftor [6] and next-generation corrector elexacaftor [7]), potentiators of conductivity and opening capacity of the ion channels (ivacaftor [8]) and their combinations (lumacaftor+ivacaftor, Orkambi; tezacaftor+ivacaftor, Symdeko; elexacaftor+tezacaftor+ivacaftor, Trikafta). Orkambi as well as Symdeko have been approved for patients with homozygous p.F508del mutation [9], while Trikafta has been approved for patients with p.F508del and with compound “minimal function mutations” [10]. Unfortunately, despite their efficacy, the drugs should be taken twice a day lifelong; in addition, they exhibit a fairly wide range of side effects that can worsen the quality of life of patients. In addition, Orkambi is effective only in the case of the homozygous p.F508del mutation, otherwise, when the p.F508del mutation occurs together with another mutation in the *CFTR* gene, the drug is ineffective [11].

Etiologically based treatment of cystic fibrosis is one of the key problems of modern medicine in the therapy of hereditary diseases, because previous studies have shown a low efficacy of classical gene therapy approaches. Gene therapy of CF has been under development since early 90s, but it displays a very low efficiency in delivering the target DNA sequence to the cells of the upper respiratory tract, despite the good results obtained in preclinical studies. Significant limitations in *CFTR* gene delivery result in no or extremely low clinical effect, which makes it impossible to use these methods in routine practice [12,13].

The development of genome editing methods, including CRISPR/Cas9, has marked the start of a new era of gene therapy for hereditary diseases. The use of so-called programmable nucleases is the basis of this group of methods [14]. Nucleases FokI (for ZFNs and TALENs methods) or Cas9 (for CRISPR/Cas9) cut the DNA at a strictly defined location in the genome [14], thus providing the opportunity to correct the mutation using one of the double strand break DNA repair methods–non-homologous end joining (NHEJ) or homology-directed repair (HDR). The latter occurs only in the presence of donor DNA which recombines with the cut DNA and allows inserting a new DNA fragment [15].

Previous attempts to correct *CFTR* mutations using ZFNs have demonstrated low efficiency of indels formation–from zero to 14%, depending on the cell culture [16–18]. The first experiments on genome editing of the p.F508del mutation in the *CFTR* gene using a donor DNA molecule have shown a lower HDR efficacy: less than 2% [17,19] or single cells that required their selection [20–25]. However, recent studies on the p.F508del mutation correction in induced pluripotent stem cells (iPSCs) [26] or in upper-airway basal stem cells [27] have shown very promising results. Both research groups have used the electroporation of Cas9 protein and guide RNA. The first group have added ssODN to the electroporation mixture and have obtained 12.7% of HDR-edited *CFTR* alleles and total 22.0% of HDR-edited cells [26], which means that at least a few cells had both alleles edited. The second group have delivered HDR template using the AAV6 vector and have achieved a 41% efficacy of the p.F508del mutation correction [27]. But despite the good results of these two studies, the average correction efficacy in other studies remains low.

The low efficacy and ongoing progress in the CRISPR/Cas9 field require the development of more specific and effective approaches to editing the p.F508del mutation. In this work, we use more specific nucleases, eSpCas9(1.1) [28] and SpCas9(HF4) [29] as well as SaCas9 [30], which have not been previously used for editing mutations in the *CFTR* gene. In addition, we use at least two sgRNAs designed to target the p.F508del mutation, which makes it possible to precisely edit only the mutant allele.

The aim of this work is to correct the p.F508del mutation in the *CFTR* gene in CFTE29o- cells and CF patient-derived iPS cells using CRISPR/Cas9.

## Materials and methods

### Plasmids and DNA donor molecules

**Fig 1:**
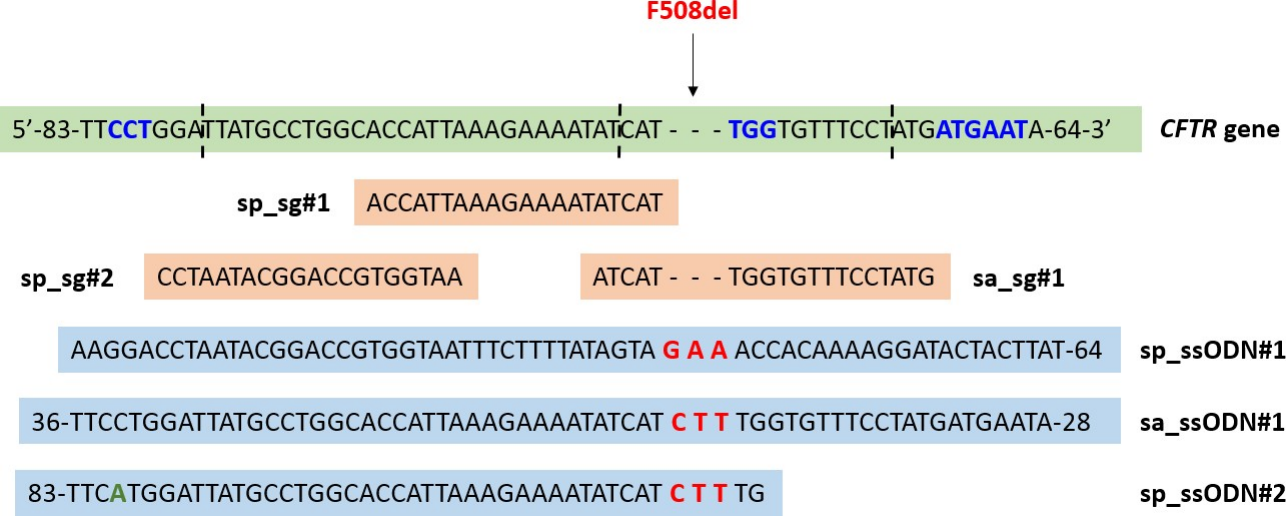
sgRNAs and ssODNs for the *CFTR* locus. The numbers in the nucleotide sequence indicate the number of nucleotides up- and downstream of the target locus. The dashed lines indicate the locations of the expected Cas9-induced DNA double strand breaks. PAMs are marked in blue (note that for the most 5’ PAM its complementary strand is marked); the nucleotides correcting the p.F508del mutation are marked in red; the silent variant in sp_ssODN#2 is marked in green. sgRNA–single guide RNA, ssODN–single-stranded oligodeoxynucleotide.

The construction of plasmids for genome editing was performed as described previously [31]. Sp_sg#1 and sa_sg#1 were manually designed to target the p.F508del mutation, while sp_sg#2 was selected to target the upstream region (Fig 1). Sp_sg#1 and sa_sg#1 do not contain the CTT sequence, so they can interact only with the mutant allele. For p.F508del mutation editing, we designed three ssODNs as described previously [32] (Fig 1). Since the cleavage sites for different sgRNAs were different, we selected three separate overlapping ssODNs–sp_ssODN#1 (for sp_sg#1), sp_ssODN#2 (for sp_sg#2) and sa_ssODN#1 (for sa-sg#1). All ssODNs were 127 nt in length, asymmetric and contained homology arms 91 nt and 36 nt from the cleavage site. Sp_ssODN#1 and sp_ssODN#2 were homologous to the target strand (the strand to which the sgRNA anneals), while sa_ssODN#1 was homologous to the opposite strand. Sp_ssODN#2 contained a silent mutation in the PAM region to prevent repeated DNA cleavage by sp_sg#2. In this work, we used four combinations of Cas9/sgRNA: Cas9(1.1)-sp_sg#1, Cas9(1.1)-sp_sg#2, Cas9(HF4)-sp_sg#1, and SaCas9-sa_sg#1. Since the efficacy of editing is presumably affected by the structure of a genomic site, sgRNAs were also tested in a synthetic plasmid pGEM-CFTR containing the *CFTR* locus (397 bp) with the p.F508del mutation.

### Reprogramming of fibroblasts, characterization of iPSCs

The study was approved by the Ethics Committee of the Research Centre for Medical Genetics (Moscow, Russia) and conducted in accordance with provisions of the Declaration of Helsinki of 1975. The patient signed an informed written consent form as an anonymous participant of the study and a donor of biological materials. IPSCs were obtained from skin fibroblasts of a male CF patient (p.F508del/p.F508del) using the CytoTune-iPS 2.0 Sendai Reprogramming Kit (Thermo Fisher Scientific, USA) according to the manufacturer's protocol. Expression of the markers of pluripotency (SSEA4, OCT4, SOX2, NANOG, TRA-1-60, TRA-1-81, OCT4, NANOG, FOXD3) was assessed by immunocytochemical analysis or RT-PCR. The karyotype analysis was performed using the GTG-differential staining method in accordance with standard cytogenetic protocols based on the International System for Human Cytogenetic Nomenclature (2016). Differentiation of iPSCs into three germ layers was performed using the embryoid body formation step and was verified by immunocytochemical staining with antibodies against b-tubulin, α-fetoprotein and fibronectin. The methods are described in more detail in S1 File.

### Genotyping

The p.F508del mutation was verified in the DNA from primary fibroblasts, iPSCs and CFTE29o- by Sanger sequencing. Genomic DNA was extracted from the cells using the Quick-gDNA Miniprep Kit (Zymo Research, USA). Direct DNA sequencing of PCR products by the Sanger method was conducted as described previously [33]. The sequences of the primers named CFTR-1 are available in S2 Table in S1 File.

### Cell cultivation and transfection

iPSCs were cultivated in Essential 8 (Thermo Fisher Scientific, USA) or TESR E8 (Stem Cell Technologies, Canada) media. Cell culture CFTE29o- (Russian Cell Culture Collection, Institute of Cytology of the Russian Academy of Sciences) was cultured in DMEM (Paneco, Russia) supplemented with 10% fetal bovine serum (PAA Laboratories, Austria), 100 U/ml penicillin, 100 μg/ml streptomycin and 4 mM L-glutamine (Paneco, Russia). Lipofection of cell culture of iPSCs was performed using the TransIT-LT1 Transfection Reagent (Mirus Bio, USA). We used 3.6 μg Cas9+sgRNA plasmid, 0.4 μg pGEM-CFTR and/or 100 pmol ssODN per 1.5×10^6^ cells. CFTE29o- cells were transfected with Lipofectamine 2000 Transfection Reagent (Thermo Fisher Scientific, USA): 5.8 μg Cas9+sgRNA plasmid, 0.6 μg pGEM-CFTR and/or 100 pmol ssODN per 3×10^5^ cells. Transfection efficacy was assessed by counting the number of fluorescent cells after transfection with 5.8 μg Cas9+sgRNA plasmid and 0.6 μg pEGFP-С1 plasmid (Clontech, USA) using flow cytometry (FloMax, Partec, Germany).

### Deep sequencing

Total DNA from transfected cells was isolated using the Quick-gDNA Miniprep Kit (Zymo Research, USA). To measure the editing efficiency in the *CFTR* gene, we performed next-generation sequencing (NGS) using a MiSeq System (Illumina, USA). According to the Illumina sequencing protocol, the required fragment size without adapter sequences should be no more than 150 bp. Thus, to separate the plasmid and genomic loci, amplification was preliminarily performed with specific primers to these loci (S2 Table in S1 File–primers CFTR-1 and pGEM-CFTR). The next step of nested PCR was performed using universal primers to the *CFTR* gene (S2 Table in S1 File–primers CFTR-2). The mean sequence coverage of the *CFTR* gene was 36,100 (ranging from 22,589 to 54,230). All variants were uploaded to the Sequence Read Archive (SRA) (https://www.ncbi.nlm.nih.gov/sra; accession no. PRJNA646350). For NGS data analysis, we used the software CRISPResso2 –Analysis of genome editing outcomes from deep sequencing data [34], which allows to obtain the efficiencies of NHEJ and HDR.

### Statistical analysis

The Kruskal-Wallis H test (ANOVA on ranks) was used to analyze differences between efficacy of plasmid and genomic loci editing. All tests were performed in STATISTICA ver. 10.0 (StatSoft, USA). The difference was considered significant if the p-value was less than 0.05.

## Results

### p.F508del editing in CFTE29o- cells

In this paper, we use the term “imperfect HDR” that means the simultaneous insertion of CTT (correction of the p.F508del mutation) and an additional change (deletion, substitution or other insertions) in the same allele; on the contrary, the term “precise HDR” means the insertion of CTT only. Hereinafter, the adjusted values (i.e. recalculated in relation to the number of transfected cells) are used.

**Fig 2:**
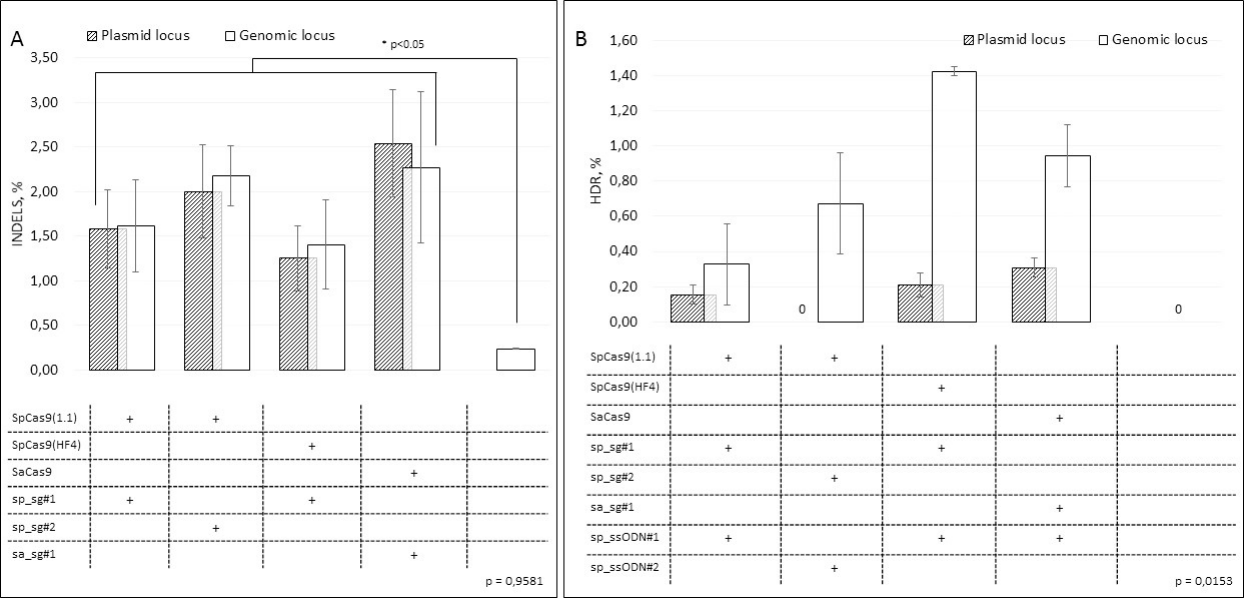
*CFTR* gene editing in CFTE29o- cells. Cells were transfected with various combinations of Cas9, sgRNA and ssODN. DNA (n = 2) was isolated 48–72 hours after transfection and the *CFTR* fragment was amplified from the genomic and plasmid loci; a sequencing library was prepared and then sequenced using a MiSeq System (Illumina); the results were analyzed using the CRISPResso2 software. A. NHEJ in the plasmid and genomic loci in the *CFTR* gene. B. HDR (p.F508del correction) in the plasmid and genomic loci in the *CFTR* gene. Results represented as mean ± SEM.

The editing efficiency of the *CFTR* gene by CRISPR/Cas9 in CFTE29o- cells was assessed in two ways. First, we analyzed the frequency of NHEJ events in the plasmid and genomic loci separately. The frequency of NHEJ did not differ (p = 0.96) between plasmid and genomic loci (Fig 2A), which means that the structure of the *CFTR* genomic locus in CFTE29o- cells does not affect the efficacy of NHEJ. The NHEJ frequency in the *CFTR* gene varied from 1.25% to 2.54% of alleles in the plasmid locus and from 1.41% to 2.27% of alleles in the genomic locus. All Cas9 and sgRNA combinations demonstrated similar activity. In untransfected cells, NHEJ frequency was significantly lower compared to experimental conditions (0.24%, p = 0.02), 92.7% of DNA changes were substitutions.

Second, we assessed HDR in the *CFTR* gene (p.F508del mutation correction) for four combinations of Cas9+sgRNA and ssODN (Fig 2B). The combinations of SpCas9(HF4)-sp_sg#1 and SaCas9-sa_sg#1 with sp_ssODN#1 tended to be more efficient than other combinations. All Cas9+sgRNA and ssODN combinations were significantly more active in genomic locus compared to the plasmid locus (p = 0.02). SpCas9(1.1)-sp_sg#2 with sp_ssODN#2 did not correct the mutation in the plasmid locus. In three out of eight cases, an imperfect HDR was observed: 9% in the combination SpCas9(1.1)-sp_sg#1 with sp_ssODN#1 in the plasmid locus, 1.5% in SpCas9(HF4)-sp_sg#1 with sp_ssODN#1 in the genomic locus, and 5% in SaCas9-sa_sg#1 with sp_ssODN#1 in the plasmid locus.

SpCas9(HF4)-sp_sg#1 together with sp_ssODN#1 were the most accurate and effective combinations in the genomic locus: the p.F508del mutation was precisely corrected in almost 50% of Cas9-induced DNA double strand breaks (Table 1).

**Table 1:** Percentage of HDR from all NHEJ events in CFTE29o- cells.

Combination of Cas9 and sgRNA	Donor DNA molecule	Percentage of HDR from all NHEJ events in the plasmid locus, %	Percentage of HDR from all NHEJ events in the genomic locus, %	Percentage of precise HDR from all NHEJ events in the plasmid locus, %	Percentage of precise HDR from all NHEJ events in the genomic locus, %
SpCas9(1.1)-sp_sg#1	sp_ssODN#1	8.89	16.83	7.73	16.83
SpCas9(1.1)-sp_sg#2	sp_ssODN#2	0	23.65	0	23.65
SpCas9(HF4)- sp_sg#1	sp_ssODN#1	14.47	50.34	14.47	49.63
SaCas9-sa_sg#1	sp_ssODN#1	10.81	29.34	10.11	29.34

sgRNA–single guide RNA, ssODN–single-stranded oligodeoxynucleotide, NHEJ–non-homologous end joining, HDR–homology-directed repair.

### P.F508del editing in iPS cells

After verification of the homozygous p.F508del mutation in the *CFTR* gene in fibroblasts from a CF patient, reprogramming was performed. The reprogramming efficiency calculated as the ratio of the number of obtained colonies to the number of cells subjected to infection was 0.2%. We confirmed expression of pluripotency markers NANOG, Tra-1-80, OCT4, Sox2, SSEA-4 and TRA-1-60 by immunofluorescence staining (Supp. Figure S1a in S1 File) and *OCT4*, *NANOG* and *FOXD3* by RT-PCR (Supp. Figure S1e in S1 File) in obtained iPSCs. The p.F508del mutation was verified by the Sanger sequencing (Supp. Figure S1c in S1 File). iPSCs showed a normal karyotype (46,XY) by GTG-differential staining method (Supp. Figure S1d in S1 File). iPSCs were capable of differentiating into three germ layers (Supp. Figure S1b in S1 File). Thus, we fully characterized the obtained iPSC line [35].

**Fig 3:**
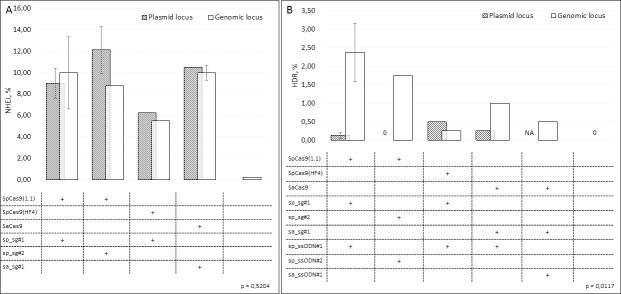
*CFTR* gene editing in iPSCs. Cells were transfected with different combinations of Cas9, sgRNA and ssODN; DNA (n = 2 or n = 1) was isolated after 48–72 hours and the *CFTR* fragment was amplified from the genomic and plasmid loci; a sequencing library was prepared and then sequenced using a MiSeq System (Illumina); the results were analyzed using the CRISPResso2 software. The Kruskal-Wallis test was used to analyze differences. A. NHEJ in the plasmid and genomic loci in the *CFTR* gene. B. HDR (p.F508del correction) in the plasmid and genomic loci in the *CFTR* gene. Results represented as mean ± SEM. iPSCs–induced pluripotent stem cells.

Similar to CFTE29o- cells, we assessed NHEJ produced by CRISPR/Cas9 in the *CFTR* gene in CF patient-derived iPSCs obtained from skin fibroblasts. We analyzed the frequency of NHEJ in the plasmid and genomic loci separately (Fig 3A). Some samples were not analyzed (N/A in the figure) or analyzed in one biological replicate due to the lack of the material for deep sequencing. As in CFTE29o- cells, the NHEJ frequency in iPSCs did not differ between plasmid and genomic loci (p = 0.5). The NHEJ frequency in the *CFTR* gene varied from 6.25% to 12.13% of alleles in the plasmid locus and from 5.5% to 10% alleles in the genomic locus. In untransfected cells, NHEJ frequency was lower compared to experimental conditions (0.22%), but difference was not significant; 87.9% of DNA changes were substitutions.

Next, we assessed CTT insertions in the *CFTR* gene (p.F508del mutation correction) for five combinations of Cas9+sgRNA and ssODN (Fig 3B). SpCas9(1.1)-sp_sg#2 with sp_ssODN#2 were not active in the plasmid locus. Similar to CFTE29o- cells, in iPSCs almost all CRISPR/Cas9 combinations were significantly more active in the genomic locus compared to plasmid one (p = 0.01). Unlike experiments with CFTE29o- cells, in iPSCs almost all used CRISPR/Cas9 combinations were precise. However, the combination SpCas9(1.1)-sp_sg#2 with sp_ssODN#2 led to imperfect HDR in all cases at the plasmid locus.

In the genomic locus, SpCas9(1.1)-sp_sg#1 together with sp_ssODN#1 tended to be the most effective combination, they were able to precisely correct 20% of Cas9-induced DNA double strand breaks (Table 2).

**Table 2:** Percentage of HDR from all NHEJ events in iPSCs.

Combination of Cas9 and sgRNA	Donor DNA molecule	Percentage of HDR from all NHEJ events in the plasmid locus, %	Percentage of HDR from all NHEJ events in the genomic locus, %	Percentage of precise HDR from all NHEJ events in the plasmid locus, %	Percentage of precise HDR from all NHEJ events in the genomic locus, %
SpCas9(1.1)-sp_sg#1	sp_ssODN#1	1.37	19.19	0	19.19
SpCas9(1.1)-sp_sg#2	sp_ssODN#2	0	16.67	0	16.67
SpCas9(HF4)- sp_sg#1	sp_ssODN#1	7.41	4.35	7.41	4.35
SaCas9-sa_sg#1	sp_ssODN#1	2.33	10.00	2.33	10.00
SaCas9-sa_sg#1	sa_ssODN#1	N/A*	4.35	N/A*	4.35

*N/A–data was not analyzed.

iPSCs–induced pluripotent stem cells, sgRNA–single guide RNA, ssODN–single-stranded oligodeoxynucleotide, NHEJ–non-homologous end joining, HDR–homology-directed repair.

## Discussion

The development of an efficient technique for correction of p.F508del mutation in the *CFTR* gene would be a major breakthrough in the treatment of cystic fibrosis. Correction of this mutation in early childhood immediately after diagnosis can help to avoid the progression of the disease, the necessity of lifelong expensive therapy and will increase the quality and duration of patient’s life. Genome editing allows targeted correction of different types of mutations, but its efficacy often remains low. In this paper, we describe the correction of the most common cause of cystic fibrosis in Europe, the p.F508del mutation, in two cell cultures homozygous for p.F508del: CFTE29o- and iPSCs.

We chose SpCas9(1.1) and SpCas9(HF4) nucleases, aiming to get in the future the best combination of high efficiency and low off-target. However, in this work we assessed only Cas9 on-target activity and its accuracy in the target locus.

In this study, we used a synthetic plasmid, pGEM-CFTR, for editing the p.F508del mutation not only in the genomic locus, but also in the plasmid to compare efficacies. Our hypothesis was that the 3D structure of the *CFTR* genomic locus in iPSCs could adversely affect the editing efficiency, since this gene is inactive in iPSCs. However, our data show that the efficiency of NHEJ in the plasmid does not differ from that in the genomic site. Thus, a synthetic plasmid can be used in *CFTR* editing experiments when cell lines are unavailable due to the rarity of mutations, as, for example, in a recent study by P. Harrison’s group [36]. As to HDR, however, in seven cases out of eight we found that the CTT insertion occurred more often only at the genomic locus, which indicates that the HDR process in exogenous and endogenous genes may be different.

In our work, the frequency of indels in the *CFTR* gene in the CFTE29o- cell culture ranged from 1.25% to 2.54% of alleles in the plasmid locus and from 1.41% to 2.27% of alleles in the genomic locus (Fig 2A). Our results are similar to the results obtained with ZFNs in similar studies in CFTE and CFBE cultures–the frequency of indels in the genome did not exceed 8% [17] and 14% [18].

In the work, we used sgRNAs which bind only the allele with the p.F508del mutation, while in the case of sgRNA which binds both mutant and wild type alleles (sp_sg#2), we used ssODN with a silent mutation in PAM for sp_sg#2, which prevents repeated DNA cleavage. Thus, we hypothesize that imperfect HDR may be the result of HDR occurring simultaneously with some other mutational events. Since the imperfect HDR is calculated as the total number of reads different from the reference one, it is possible that some of the registered mutations are sequencing errors which appeared in 1 nucleotide per 1 read. The relatively high level of NHEJ events in control untransfected CFTE29o- and iPS cells, in our opinion, can also be explained by sequencing errors, since the vast majority of changes in DNA are represented by unique substitutions each found in a single read, which are a characteristic drawback of the Illumina NGS platform [37]. At the same time, no HDR events were observed in untransfected CFTE29o- and iPS cells.

The HDR frequency in the CFTE29o- cells in our work varied for different combinations of Cas9/sgRNA/ssODN. The combinations of SpCas9(HF4)-sp_sg#1 and SaCas9-sa_sg#1 with sp_ssODN#1 tended to be 3–7 times more active in the genomic locus in the CFTE29o- cells compared to the plasmid locus and had a low percentage of imperfect HDR in the genomic locus (Fig 2B). The cumulative HDR frequency in the genomic locus for these two combinations was 1.42% and 0.94% of alleles, respectively. This result is in good agreement with previously published studies, in which the efficiency of the correction of the p.F508del mutation in CFTE cells has not exceeded 2% [17,19]. We showed that almost 50% of *CFTR* alleles cleaved by SpCas9(HF4)-sp_sg#1 were precisely repaired by sp_ssODN#1 in the CFTE29o- cells.

Induced pluripotent stem cells are poorly correctable by genome editing methods, especially in the case of inactive genes [38]. Almost all published papers described a low efficacy of the p.F508del correction in iPSCs by TALENs [20,21] or CRISPR/Cas9 [23,36] methods. Knowing that inactive genes are less amenable to genome editing, we used a targeted plasmid with a fragment of the *CFTR* gene with the p.F508del mutation to compare the efficiency of editing in the plasmid and in the genome. However, there were no differences in the editing efficiency (NHEJ) of the genomic and plasmid loci (Fig 3A). The NHEJ frequency in the *CFTR* gene in iPSCs varied from 6.25% to 12.13% of alleles in the plasmid locus and from 5.5% to 10% of alleles in the genomic locus.

The HDR efficacy in the genomic locus of the *CFTR* gene was higher in the case of SpCas9(1.1)-sp_sg#1 with sp_ssODN#1 (2.38% of corrected alleles), the percentage of precise HDR was also high (100% of corrected alleles) (Table 2). Unlike CFTE29o- cells, in iPSCs only 20% of alleles cleaved by SpCas9(1.1)-sp_sg#1 were repaired by precise HDR. Our results demonstrate a lower HDR efficiency in the *CFTR* gene in iPSCs compared to recent studies using RNPs in stem cells [26,27]. The delivery of CRISPR/Cas9 as an RNP complex for the *CFTR* gene (and particularly for the correction of the p.F508del mutation) is likely to be more efficient, which can lead to an increase in editing efficiency.

Correction of the p.F508del mutation in iPSCs using CRISPR/Cas9 is indeed a very rare event and occurs in single cells. Our results show that it is difficult or even impossible to carry out such manipulations with cells without a subsequent selection, which has been confirmed by other researchers [23,36].

## Conclusion

Our results demonstrate that though CRISPR/Cas9 can be used to correct single cells in the culture, it is still a long way off to achieve clinically significant results. However, genome editing in iPSCs followed by cell sorting, cultivation and differentiation can be used for the development of the combined genome editing and cell transplantation therapy using patient’s own cells. Autologous transplantation of edited cells allows to avoid cell rejection and graft versus host-disease problems.

## Supporting information

S1 File (DOCX)
