# Supplementary material for: P.F508del editing in cells from cystic fibrosis patients
Source: PLoS One. 2020 Nov 11;15(11):e0242094. doi: 10.1371/journal.pone.0242094 (PMC7657551; doi:10.1371/journal.pone.0242094)
Supplement: S1 File — (DOCX) [file pone.0242094.s001.docx]

**Supplementary materials**

**P.F508del editing in cells from cystic fibrosis patients**

Svetlana A. Smirnikhina^1,*^, Ekaterina V. Kondrateva^1^, Elmira P. Adilgereeva^1^, Arina A. Anuchina^1^, Milyausha I. Zaynitdinova^1^, Yana S. Slesarenko^1^, Angelina S. Ershova^2^, Kirill D. Ustinov^1^, Matvei I. Yasinovsky^1^, Elena L. Amelina^3^, Ekaterina S. Voronina^1^, Valentina D. Yakushina^1^, Vyacheslav Yu. Tabakov^1^, Alexander V. Lavrov^1^

Supplementary Materials and Methods

***Reprogramming of fibroblasts***

The study was approved by the Ethics Committee of the Research Centre for Medical Genetics (Moscow, Russia) and conducted in accordance with provisions of the Declaration of Helsinki of 1975. The primary skin fibroblast culture from a male CF patient (p.F508del/p.F508del) was obtained from an inner forearm skin biopsy material. The primary fibroblasts were cultivated in the Amniokar proliferative medium (Paneco, Russia). Then the obtained cultures were deposited and are now available at the Centre for Collective Use “Biobank” (Research Centre for Medical Genetics, Moscow, Russia). All cultivation and sample preparation services were provided by “Biobank”. The patient signed an informed written consent form as an anonymous participant of the study and a donor of biological materials. The primary fibroblast culture frozen in the second passage was thawed in a fibroblast culture medium (DMEM + GlutaMax (Life Technologies, USA), 1% amino acid mixture MEM (Life Technologies, USA), 5.5 mM β-mercaptoethanol (Sigma-Aldrich, USA), penicillin-streptomycin (50 U/ml; 50 μg/ml) (Paneco, Russia)) with the addition of 10% HyClone fetal bovine serum (FBS) (GE Healthcare Life Sciences, USA) and seeded into a 25 cm^2^ culture flask (Corning Costar, USA). After the cells reached confluence of 95-100%, they were transferred to 6-well culture plates (Corning Costar, USA) at a density of 2-8×10^4^ cells per well. Reprogramming was performed using the CytoTune-iPS 2.0 Sendai Reprogramming Kit (Thermo Fisher Scientific, USA) according to the manufacturer's protocol. Cells were cultured in the fibroblast culture medium supplemented with 10% FBS. On day 8 after reprogramming, the cells were transferred to culture plates (Corning Costar, USA) pre-coated with vitronectin (Thermo Fisher Scientific, USA) according to the manufacturer’s protocol. One day after reseeding, cells were transferred to Essential 8 Medium (Thermo Fisher Scientific, USA). On the 10-12th day, the appearance of the first colonies was observed. By the 28th day after reprogramming, the colonies were mechanically isolated and cultured.

***Characterization of iPSCs***

Expression of markers SSEA4, OCT4, SOX2, NANOG, TRA-1-60, TRA-1-81 was assessed by immunocytochemical analysis with antibodies in accordance with the manufacturer's instructions (Supplementary Table S1). Immunofluorescence was registered using the Axio VERT A1 fluorescence microscope (Zeiss, Germany) and ZEN software. Expression of *OCT4*, *NANOG,* *FOXD3, B2M* and *GAPDH* was assessed by RT-PCR. Total RNA was isolated from cells using the RNeasy Plus mini kit (Qiagen, Germany). The reverse transcription reaction was performed using 1 μg of total RNA per reaction, random primers (DNA-Synthesis, Russia), M-MLV reverse transcriptase (Promega, USA), ribonuclease inhibitor (Promega, USA), nucleotide mixture (Fermentas, USA) according to the manufacturer’s instructions. PCR amplification of the reaction products was performed using SYBR Green I in CFX96 (BioRad, USA). List of primers is presented in Supplementary Table S2. The karyotype analysis was performed using the GTG-differential staining method in accordance with standard cytogenetic protocols based on the International System for Human Cytogenetic Nomenclature (2016).

***In vitro differentiation of iPSCs***

Differentiation of iPSCs into three germ layers was performed using the embryoid body formation step. The iPSC colonies were detached from the plate surface using Versen's solution (Paneco, Russia) according to a standard protocol, dissociated into fragments in 1 ml of the TeSR-E8 medium (StemCell Technologies, Canada) with 5 μM Y27632 (StemCell Technologies, Canada) and seeded into a 6-well Corning Costar Ultra-Low Attachment plate (Corning Costar, USA). After 3 days, the medium was replaced with a fresh one consisting of 1⁄2 volume of the TeSR-E8 medium and 1⁄2 volume of the ES medium (composition: DMEM/F12, 20% KO Serum replacement, 2 mM L-glutamine, 1% mixture of amino acids (all from Thermo Fisher Scientific, USA), 0.1 mM β-mercaptoethanol (Sigma-Aldrich, USA), penicillin-streptomycin (50 U/ml; 50 μg/ml) (Paneco, Russia)). After another 3 days, half of the volume of the medium in the wells was replaced with the culture medium for embryoid bodies (EB medium), consisting of 1–2 volumes of the ES medium and 1–2 volumes of the fibroblast culture medium without FBS. Then, a replacement of 1–2 volumes of medium in the well was carried out once every 2–3 days for 14 days, while FBS (GE Healthcare Life Sciences, USA) was injected and, with each subsequent change, its content in the fresh medium was gradually increased from 1% to 10% of total medium. On the 16th day, the embryoid bodies were carefully transferred to gelatin-coated (Sigma-Aldrich, USA) 60 mm Petri dishes (SPL Life Sciences, Korea) in the culture medium for embryoid bodies supplemented with 10% FBS. On the 30th day of cultivation, the cells were fixed in 4% paraformaldehyde (Sigma-Aldrich, USA) and immunocytochemistry was performed for cell markers belonging to three germ layers (b-tubulin, α-fetoprotein and fibronectin, Supplementary Table S1).

***Genotyping***

The p.F508del mutation was verified in DNA from primary fibroblasts, iPSCs and CFTE29o- cells by Sanger sequencing. Genomic DNA was extracted from the cells using the Quick-DNA Miniprep Kit (Zymo Research, USA). Direct DNA sequencing of PCR products by the Sanger method was conducted as described previously [30]. The sequences of the primers named CFTR-1 are available in Supplementary Table S2.

Supplementary Table S1. List of used antibodies for iPSC characterization

| Antibodies | Isotype | Species | Vendor | Cat. # |
| --- | --- | --- | --- | --- |
| Primary Antibodies | | | | |
| Anti-SSEA4 | IgG3 | Mouse | Thermo Fisher Scientific, Germany | 414000 |
| Anti-SOX2 | IgG1 | Mouse | Abcam, USA | ab79351 |
| Anti-TRA-1-60 | IgM | Mouse | Thermo Fisher Scientific, Germany | 411000 |
| Anti-TRA-1-81 | IgM | Mouse | Thermo Fisher Scientific, Germany | 411100 |
| Anti-Oct4 | IgG | Rabbit | Abcam, USA | ab18976 |
| Anti-Nanog | IgG | Rabbit | Thermo Fisher Scientific, Germany | PA1-097X |
| Anti-beta Tubulin | IgG1 | Mouse | Abcam, USA | ab131205 |
| Anti-alpha 1 Fetoprotein | IgG1 | Mouse | Abcam, USA | ab3980 |
| Anti-Fibronectin | IgG | Rabbit | Abcam, USA | ab2413 |
| Secondary Antibodies | | | | |
| Anti-Mouse IgG (H+L), Alexa Fluor 555 | IgG | Goat | Thermo Fisher Scientific, Germany | A21422 |
| Anti-Mouse IgG (H+L), Alexa Fluor 488 | IgG | Goat | Thermo Fisher Scientific, Germany | A11029 |
| Anti-Rabbit IgG (H+L), Alexa Fluor 488 | IgG | Donkey | Thermo Fisher Scientific, Germany | A21206 |

Supplementary Table S2. List of primers for PCR and RT-PCR

| **Gene** | **Primers** | **Product length, bp** |
| --- | --- | --- |
| ***OCT4*** | F-5’-CGACCATCTGCCGCTTTGAG-3’ | 588 |
|  |  |  |
|  | R-5’-CCTAGCTCCTCCCCTCCCCCTGTC-3’ |  |
|  |  |  |
| ***NANOG*** | F-5’-CAGCCCTGATTCTTCCACCAGTCCC-3’ | 343 |
|  |  |  |
|  | R-5’-TGGAAGGTTCCCAGTCGGGTTCACC-3’ |  |
|  |  |  |
| ***B2M*** | F-5’-CTGCCGTGTGAACCATGTGA-3’ | 103 |
|  |  |  |
|  | R-5’-CAATCCAAATGCGGCATCTTC-3’ |  |
|  |  |  |
| ***FOXD3*** | F-5’-CAAGCCCAAGAACAGCCTAGTGAA-3’ | 203 |
|  |  |  |
|  | R-5’-TGACGAAGCAGTCGTTGAGTGAGA-3’ |  |
|  |  |  |
| ***GAPDH*** | F-5’-GCTCTCTGCTCCTCCTGTTC-3’ | 115 |
|  |  |  |
|  | R-5’-ACGACCAAATCCGTTGACTC-3’ |  |
|  |  |  |
| ***CFTR-1 (genomic)*** | F-5’-TGCATAGCAGAGTACCTGAAACAGGA-3’ | 500 (mut)/503 (wt) |
|  |  |  |
|  | R-5’-TTGATCCATTCACAGTAGCTTACCCA-3’ |  |
|  |  |  |
| ***pGEM-CFTR*** | F-5'-CCATGGCCGCGGGATTAA-3' | 491 |
|  |  |  |
|  | R-5'-GAATACTCAAGCTATGCATCCAACG-3' |  |
|  |  |  |
| ***CFTR-2 (for NGS)*** | F-5'-TGGAGCCTTCAGAGGGTAAAAT-3' | 145 |
|  |  |  |
|  | F-5'-GCTTTGATGACGCTTCTGTATCT-3' |  |
|  |  |  |


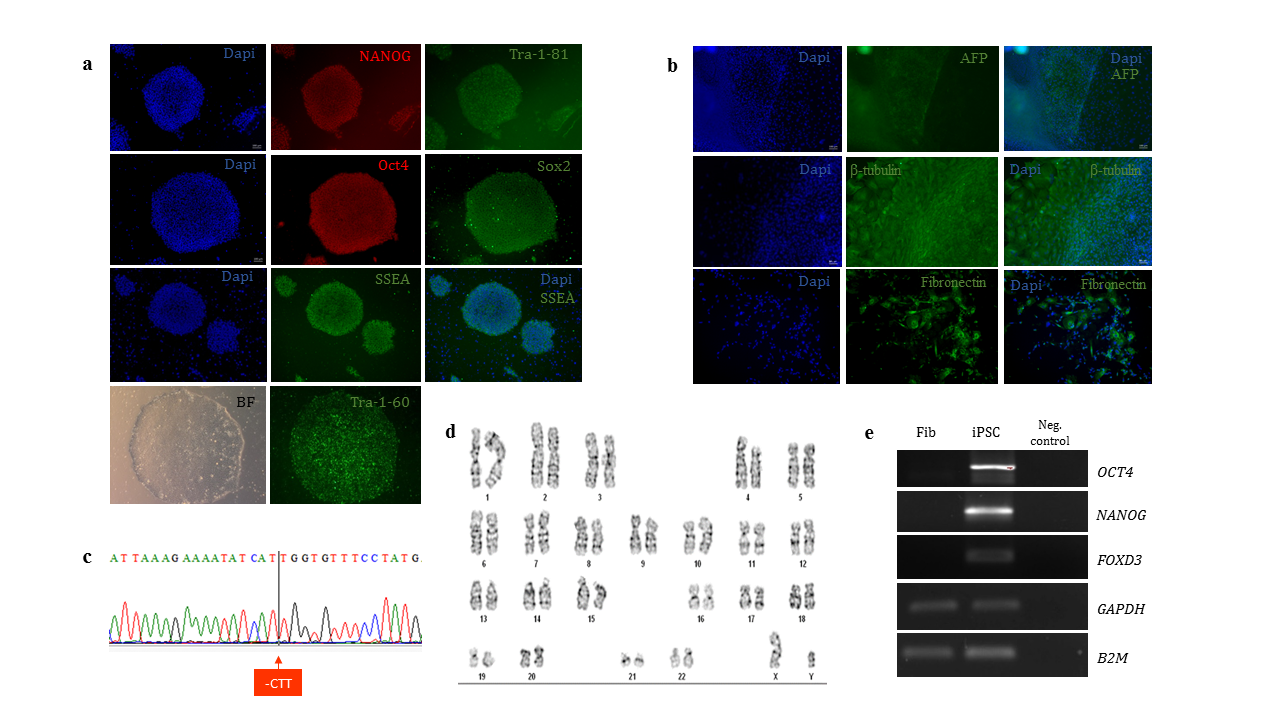


Supplementary Figure S1. iPSC characterization. (a) Immunofluorescence staining for pluripotency markers NANOG, Tra-1-80, OCT4, Sox2, SSEA-4 and TRA-1-60 in iPSCs. Nuclei were stained with DAPI (blue). BF, bright field. Magnification x100. (b) Immunostaining of iPSC derivatives on day 30 of differentiation revealed expression of endodermal (AFP), ectodermal (β-Tubulin) and mesodermal (Fibronectin) marker proteins. Nuclei were stained with DAPI (blue). Magnification x100. (c) Sequencing confirmed the patient-specific p.F508del mutation in iPSCs. (d) iPSCs showed a normal karyotype (46,XY). (e) OCT4, NANOG and FOXD3 were expressed in iPSCs, but not in patient’s fibroblasts by RT-PCR. iPSCs – induced pluripotent stem cells, AFP – alpha-fetoprotein, Fib – fibroblasts, Neg. control – negative control.


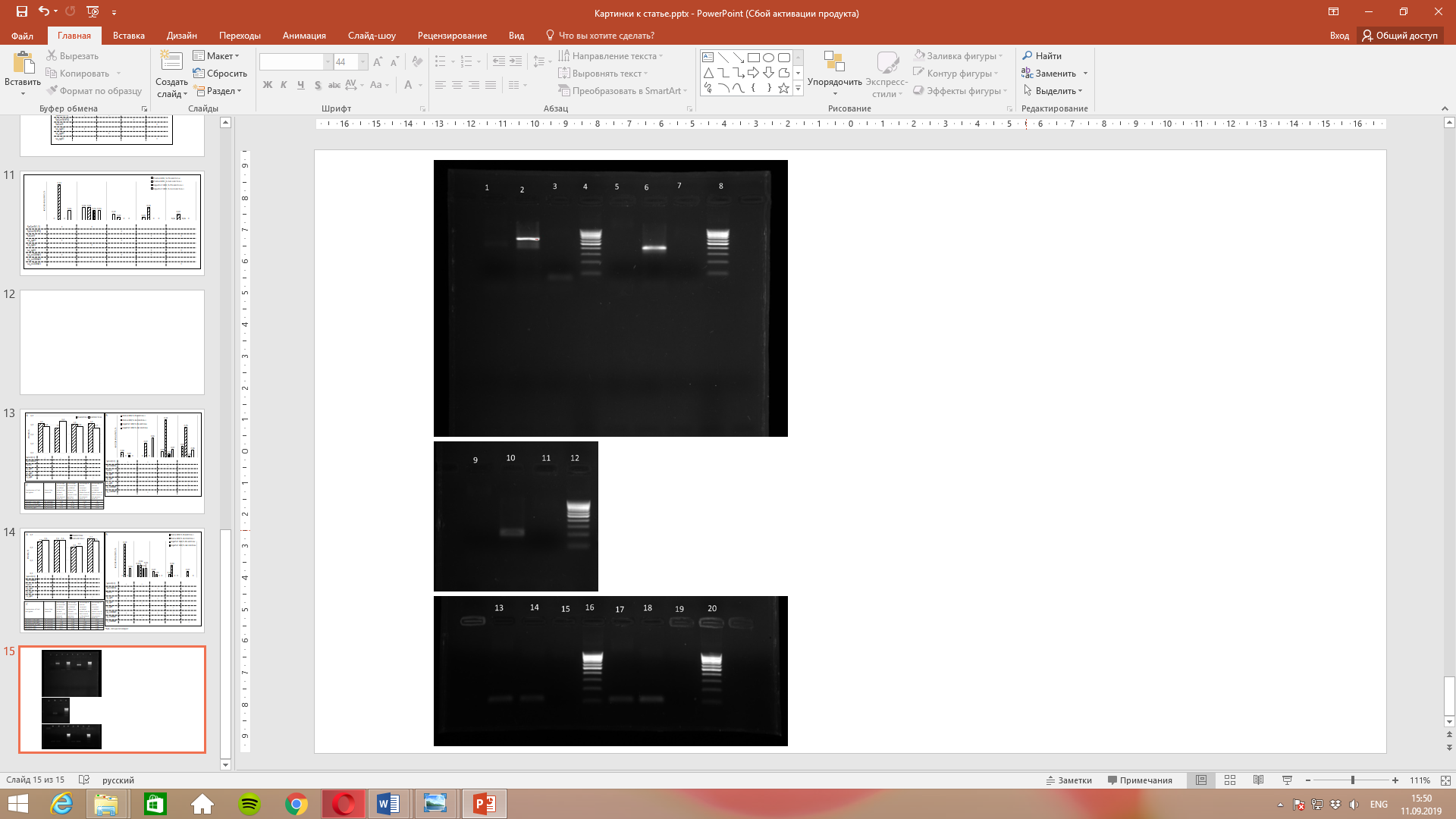


Supplementary Figure S2. Agarose (1.8%) gel electrophoresis of RT-PCR products (additional gel image for Supplementary Figure S1). Ethidium Bromide staining.

1 – *OCT4* in fibroblasts

2 – *OCT4* in iPSCs (588 bp)

3 – Negative control for *OCT4* (no DNA added)

4 – 100 bp Ladder

5 – *NANOG* in fibroblasts

6 – *NANOG* in iPSCs (343 bp)

7 – Negative control for *NANOG* (no DNA added)

8 – 100 bp Ladder

9 – *FOXD3* in fibroblasts

10 – *FOXD3* in iPSCs (203 bp)

11 – Negative control for *FOXD3* (no DNA added)

12 – 100 bp Ladder

13 – *GAPDH* in fibroblasts (115 bp)

14 – *GAPDH* in iPSCs (115 bp)

15 – Negative control for *GAPDH* (no DNA added)

16 – 100 bp Ladder

17 – *B2M* in fibroblasts (103 bp)

18 – *B2M* in iPSCs (103 bp)

19 – Negative control for *B2M* (no DNA added)

20 – 100 bp Ladder
